# Relationship between renal function and blood pressure dipping status in renal transplant recipients: a longitudinal study

**DOI:** 10.1186/s12882-021-02523-7

**Published:** 2021-09-30

**Authors:** David A. Jaques, Patrick Saudan, Chantal Martinez, Axel Andres, Pierre-Yves Martin, Antoinette Pechere-Bertschi, Belen Ponte

**Affiliations:** 1 Division of Nephrology and Hypertension, Geneva University Hospitals, Rue Gabrielle-Perret-Gentil 4, 1205 Geneva, Switzerland; 2 Division of Transplantation and Visceral Surgery, Geneva University Hospitals, Geneva, Switzerland

**Keywords:** Ambulatory blood pressure monitoring, Dipping status, Glomerular filtration rate, Office blood pressure, Renal transplant

## Abstract

**Background:**

Hypertension (HT) is associated with adverse outcomes in kidney transplant (KTX) recipients. Blunting of physiological decrease in nighttime compared to daytime blood pressure (non-dipping status) is frequent in this setting. However, weather non-dipping is independently associated with renal function decline in KTX patients is unknown.

**Methods:**

We retrospectively screened KTX outpatients attending for a routine ambulatory blood pressure monitoring (ABPM) (T1) at a single tertiary hospital. Patients had two successive follow-up visits, 1 (T2) and 2 (T3) years later respectively. Routine clinical and laboratory data were collected at each visit. Mixed linear regression models were used with estimated glomerular filtration rate (eGFR) as the dependent variable.

**Results:**

A total of 123 patients were included with a mean follow-up of 2.12 ± 0.45 years after ABPM. Mean age and eGFR at T1 were 56.0 ± 15.1 and 54.9 ± 20.0 mL/min/1.73m^2^ respectively. 61 patients (50.4%) had sustained HT and 81 (65.8%) were non-dippers. In multivariate analysis, systolic dipping status was positively associated with eGFR (*p* = 0.009) and compared to non-dippers, dippers had a 10.4 mL/min/1.73m^2^ higher eGFR. HT was negatively associated with eGFR (*p* = 0.003).

**Conclusions:**

We confirm a high prevalence of non-dippers in KTX recipients. We suggest that preserved systolic dipping is associated with improved renal function in this setting independently of potential confounders, including HT and proteinuria. Whether modification of dipping status by chronotherapy would preserve renal function remains to be tested in clinical trials.

**Supplementary Information:**

The online version contains supplementary material available at 10.1186/s12882-021-02523-7.

## Background

Ambulatory blood pressure monitoring (ABPM) allows serial blood pressure (BP) measurements over a 24-h period. Compared to office BP measurement (OBPM), ABPM is a stronger predictor of target organ damage (TOD), cardiovascular (CV) events as well as all-cause and CV mortality in hypertensive patients [1, 2]. The normal BP profile follows a circadian pattern where night-time values are at least 10% lower than daytime values (dipping status). Blunting or absence of this physiologic phenomenon (non-dipping status) is common in chronic kidney disease (CKD) and has been associated with decline of kidney function and progression of CV diseases [3–5]. Whether dipping status is correlated to adverse outcome independently of hypertension (HT) and other confounders is debated as various studies showed conflicting results [6–8].

HT is a frequent disorder in kidney transplant (KTX) recipients and is regarded as a major risk factor for CV disease, chronic allograft nephropathy and graft loss [9–11]. As in the general population, HT is frequently misclassified in KTX patients and ABPM has proved to be a valuable tool in detecting white-coat HT, masked HT and non-dipping status, which are highly prevalent after successful transplantation [12–14]. Some studies previously reported on the relationship between circadian BP pattern and kidney function in KTX patients [15–17]. However, cross-sectional design and lack of adjustment for potential confounders such as BP values and proteinuria hampered definitive conclusions. Recently, the longitudinal relationship between baseline ABPM readings and evolution of estimated glomerular filtration rate (eGFR) over a 3.7 year follow-up has been investigated in 260 KTX recipients [18]. Authors showed that 24 h, daytime and night-time SBP were negatively associated with eGFR decline. However, circadian BP patterns were not considered and the evolution of eGFR over time was not specifically described.

Based on current state of knowledge, we conducted a longitudinal cohort study in order to i) characterize circadian BP patterns measured by ABPM in KTX patients, ii) compare OBPM and ABPM readings in this population and iii) describe the evolution of eGFR over time and its relationship with dipping status independently of potential confounders.

## Methods

### Study design

We retrospectively screened adult KTX patients between 2003 and 2016 at a single tertiary hospital. During this time period, 401 adults had a renal transplant. Of those, 140 (34.9%) had ABPM performed as part of their routine follow-up. Inclusion criteria were i) ABPM available after KTX, ii) a minimum of 2-year follow-up after ABPM and iii) age ≥ 18. Exclusion criteria were i) unwilling to participate, ii) multi-organ graft, iii) haemodialysis or peritoneal dialysis and iv) pregnancy. At our institution, post-transplant ABPM are not routinely ordered but are instead requested by the attending physician based on individual clinical appreciation.

Variables were longitudinally collected at four distinct visits: T0 corresponded to discharge after transplant, T1 corresponded to ABPM while T2 and T3 corresponded to subsequent follow-up visits, 1 and 2 years after ABPM respectively. The time interval between T0 and T1 was not predefined. The time interval between T1 and T2 as well as T2 and T3 was 1 year. Thus, all included patients had a 2-year follow-up.

### Variables collection

All patients underwent a 24 h ABPM using a DIASYS INTEGRA II monitor at T1. Cuff size was chosen based on arm circumference. BP was recorded every 15 min from 7 am to 10 pm and every 30 min from 10 pm to 7 am. Daytime and night-time periods were defined by patient themselves according to their own daily schedule. Measures validity intervals were as follows: Systolic BP (SBP) > 50 mmHg and diastolic BP (DBP) > 30 mmHg and < 150 mmHg [5].

Demographic characteristics, medical history, current medication, OBPM and laboratory data were collected at T0, T1, T2 and T3. Serum creatinine was analysed by standard clinical laboratory method. Proteinuria was estimated on a single urinary specimen. OBPM was measured by oscillometric sphygmomanometer in the seated position after a resting period of ≥ 5 min according to European guidelines [19].

Renal ultrasound (US) was performed by experimented radiologists as part of routine follow-up in a subset of patients. Renal resistive index (RRI) were measured in three segmental arteries (superior, middle and inferior).

### Definitions

HT related definition were based on latest European guidelines [19]. Thus, HT on ABPM was defined as 24-h SBP ≥ 130 and/or DBP ≥ 80 mmHg. HT on OBPM was defined as SBP ≥ 140 and/or DBP ≥ 90 mmHg. The terms “white-coat”, “masked” and “sustained” HT were used in untreated as well as treated patients. Dipping and non-dipping statuses were defined as BP night-time decline of ≥ 10% and < 10% compared to daytime values, respectively and independently of the presence of HT. eGFR was estimated by the CKD-EPI equation using IDMS measured creatinine [20]. In statistical analyses, the presence HT was defined exclusively by related medication, unless otherwise specified. The presence of diabetes and dyslipidaemia were defined by related medication.

### Statistical analysis

Continuous variables are expressed as mean ± standard deviation (SD) or median (interquartile range) according to distribution and categorical variables as number and relative frequencies (%). Normality of distribution was assessed graphically. No outliers were predefined. Variables were compared between groups (dipping vs non-dipping status as well as low eGFR vs high eGFR) using Student’s T test and Chi2 for continuous and categorical variables respectively.

First, multivariate linear regressions were used with eGFR at T1 as the dependent variable and systolic dipping status as the main independent variable. The following covariates were a priori selected as potential confounders, based on prior scientific knowledge: gender, age, body mass index (BMI), smoking, HT, diabetes, graft vintage (corresponding to the timespan between T0 and T1), proteinuria, donor type (deceased vs living), past rejection, use of calcineurin inhibitors, use of steroid and use of renin angiotensin aldosterone blocker. Then, in longitudinal analysis, we considered repeated measures over time (T1, T2, T3) and implemented multi-level mixed effect analysis to account for inter-dependence of data. The same above specified covariates were used in longitudinal models. Patient identification was considered as the grouping variable while random effects were applied to time variable and intercept. Interaction between ABPM parameter and time was considered in every model. Modification effect was considered significant if *p*-value for likelihood ratio test (LRT) comparing models with and without interaction term was < 0.1

Sensitivity analyses were conducted considering i) HT defined by ABPM values instead of related medication, ii) diastolic and mean dipping status instead of systolic dipping status and iii) systolic dip as continuous variable instead of systolic dipping status as a binary variable.

For every model, linearity of relationship, normality of residuals and homoscedasticity of residuals were assessed graphically. Collinearity was assessed using the variance inflation factors method. Log-normal variables were log-transformed when used in regression models if necessary.

Data were considered to be missing completely at random and therefore patients with any missing value were excluded from the multivariate analyses. For every model, results are presented as β coefficients and associated 95% confidence intervals (95% CI) as well as *p*-values. A two-sided *p*-value < 0.05 was considered significant. Statistical analyses were conducted using STATA version 15 (StataCorp, 4905 Lakeway Drive, College Station, Texas 77845 USA) [21].

## Results

Between 2003 and 2016, 401 adults had a renal transplant. Of those, 140 (34.9%) had ABPM performed as part of their routine follow-up. We excluded 17 patients: Eight for undocumented follow-up, one for unavailable night-time ABPM, one for insufficient ABPM quality and four because of missing values on ABPM. Finally, three patients were excluded as they required haemodialysis. Thus 123 (87.8%) patients without missing values on ABPM were included in the present analysis. Compared to renal transplant patients who had an ABPM performed, those who did not have an ABPM performed had similar mean age and proportion of men: 52.8 ± 15.0 vs 50.7 ± 15.4, *p* = 0.20 and 63.0 vs 59.1%, *p* = 0.45 respectively.

Among included patients, 21 (17.3%) had no HT on OBPM or ABPM, 13 (10.7%) had white-coat HT, 26 (21.4%) had masked HT and 61 (50.4%) had sustained HT. Non-dipping status was present in 81 (65.8%) of patients.

Patient’s characteristics according to systolic dipping status at the time of ABPM (T1) are described in Table 1. Median graft vintage at ABPM (corresponding to the timespan between T0 and T1) was 2.5 (0.7 – 6.0) years. Compared to the non-dipping group, dippers had higher eGFR, lower prescription rate of CNI and lower tacrolimus trough levels (*p* < 0.05 for all). Patients tended to be younger and to have longer graft vintage in the dipping group without reaching statistical significance. Other considered characteristics were similar between groups.

**Table 1:** Patients characteristics according to systolic dipping status at T1

**Characteristics**	**Overall (*N* = 123)**	**Non-dipping (*N* = 81)**	**Dipping (*N* = 42)**	***P* value**
**Clinical characteristics**				
**Age (years)**	56.0 ± 15.1	57.8 ± 14.4	52.7 ± 16.0	0.07
**BMI (kg/m**^**2**^**)**	25.7 ± 4.0	26.1 ± 4.2	25.0 ± 3.6	0.21
**Gender (female)**	46 (37.4%)	30 (37.0%)	16 (38.1%)	0.90
**Smoking**	21 (17.0%)	12 (14.8%)	9 (21.4%)	0.35
**Diabetes**^**a**^	28 (22.9%)	21 (26.2%)	7 (16.6%)	0.23
**Hypertension**^**a**^	104 (85.2%)	70 (87.5%)	34 (80.9%)	0.33
**Dyslipidemia**^**a**^	70 (57.3%)	49 (61.2%)	21 (50.0%)	0.23
**IHD**	17 (13.9%)	14 (17.2%)	3 (7.3%)	0.13
**PVD**	12 (9.8%)	9 (11.1%)	3 (7.3%)	0.50
**OSA**	17 (13.8%)	14 (17.2%)	3 (7.1%)	0.12
**Paraclinical characteristics**				
**eGFR (mL/min/1.73m**^**2**^**)**	54.9 ± 20.0	51.7 ± 19.1	61.1 ± 20.3	**0.012**
**Proteinuria (g/day)**	0.1 (0.1 – 0.3)	0.1 (0.1 – 0.2)	0.2 (0.1 – 0.4)	0.68
**RRI (%)**	67.6 ± 7.9	67.3 ± 7.8	68.2 ± 8.2	0.64
**Transplant characteristics**				
**Graft vintage**^**b**^** (years)**	2.5 (0.7 – 6.0)	2.0 (0.6 – 5.0)	3.0 (0.9 – 8.7)	0.05
**Deceased donor**	66 (54.1%)	44 (54.3%)	22 (53.6%)	0.94
**Past rejection**	22 (17.8%)	13 (16.0%)	9 (21.4%)	0.46
**Pre-transplant status**				
- **Pre-emptive**	28 (22.7%)	19 (23.4%)	9 (21.4%)	0.41
- **PD**	14 (11.3%)	7 (8.6%)	7 (16.6%)	
- **HD**	81 (65.8%)	55 (67.9%)	26 (61.9%)	
**Medication**				
**CNI**	117 (95.1%)	80 (98.7%)	37 (88.1%)	**0.009**
**Prednisone**	76 (66.0%)	54 (70.1%)	22 (57.8%)	0.19
**RAA blocker**^**c**^	44 (36.0%)	26 (32.1%)	18 (43.9%)	0.20
**FK level (ng/mL)**^**d**^	8.1 ± 3.0	8.5 ± 3.2	7.1 ± 2.0	**0.039**
**CsA level (ng/mL)**^**e**^	136.1 ± 39.1	138.1 ± 46.5	131.4 ± 11.2	0.75

*Abbreviations*: *BMI* Body mass index, *IHD* Ischemic heart disease, *PVD* Peripheral vascular disease, *OSA* Obstructive sleep apnoea, *eGFR* Estimated glomerular filtration rate, *RRI* Renal resistive index, *PD* Peritoneal dialysis, *HD* Haemodialysis, *CNI* Calcineurin inhibitor, *RAA* Renin angiotensin aldosterone, *FK* Tacrolimus, *CsA* Cyclosporine

^a^Defined based on related medication

^b^Corresponding to the timespan between T0 and T1

^c^Angiotensin converting enzyme inhibitor or angiotensin II receptor blocker

^d^Available in a subgroup of 96 patients

^e^Available in a subgroup of 17 patients

ABPM readings according to systolic dipping status at T1 are described in Table 2. Systolic dip was 0.6 ± 6.9% in non-dippers and 14.5 ± 3.5% in dippers. Preserved systolic dipping status was associated with preserved diastolic and mean dipping statuses (*p* < 0.001 for both). Pearson correlation coefficients between systolic dippers and diastolic dippers as well as between systolic dippers and mean dippers were 62.7 and 82.9% respectively. Compared to the non-dipping group, dippers had higher diurnal MAP (*p* = 0.047) and lower nocturnal SBP, DBP and MAP (*p* < 0.001 for all). Prevalence of HT was similar between groups.

**Table 2:** ABPM readings according to systolic dipping status at T1

**Characteristics**	**Overall (*N* = 123)**	**Non-dipping (*N* = 81)**	**Dipping (*N* = 42)**	***P* value**
**24-h SBP mmHg**	131.5 ± 14.1	131.4 ± 14.2	131.7 ± 14.2	0.89
**24-h DBP mmHg**	81.2 ± 11.0	81.1 ± 11.5	81.5 ± 10.3	0.88
**24-h MBP (mmHg)**	97.0 ± 13.2	96.3 ± 14.9	98.4 ± 9.2	0.41
**Diurnal SBP (mmHg)**	134.0 ± 14.6	132.3 ± 14.6	137.2 ± 14.2	0.08
**Diurnal DBP (mmHg)**	83.3 ± 11.3	82.7 ± 11.5	84.5 ± 10.9	0.42
**Diurnal MBP (mmHg)**	99.6 ± 10.4	98.2 ± 10.7	102.2 ± 9.2	**0.047**
**Nocturnal SBP (mmHg)**	126.5 ± 16.3	131.3 ± 16.1	117.1 ± 12.3	** < 0.001**
**Nocturnal DBP (mmHg)**	77.2 ± 10.4	79.5 ± 10.1	72.8 ± 9.5	** < 0.001**
**Nocturnal MBP (mmHg)**	93.2 ± 10.5	96.1 ± 10.4	87.7 ± 8.5	** < 0.001**
**Systolic dip (%)**	5.4 ± 8.8	0.6 ± 6.9	14.5 ± 3.3	** < 0.001**
**Diastolic dip (%)**	6.9 ± 8.4	3.5 ± 7.3	13.5 ± 6.2	** < 0.001**
**Mean dip (%)**	6.1 ± 8.2	1.9 ± 6.7	14.1 ± 3.2	** < 0.001**
**Diastolic dipping**	45 (36.5%)	12 (14.8%)	33 (78.5%)	** < 0.001**
**Mean dipping**	41 (35.3%)	5 (6.5%)	36 (90.0%)	** < 0.001**
**Hypertension on ABPM**	89 (72.3%)	57 (70.3%)	32 (76.1%)	0.49

*Abbreviations*: *SBP* Systolic blood pressure, *DBP* Diastolic blood pressure, *MBP* Mean blood pressure, *ABPM* Ambulatory blood pressure monitoring

Median value of eGFR at T1 was 52.6 mL/min/1.73m^2^ in 126 patients without missing values on eGFR. Patient’s characteristics according to median eGFR value at T1 are described in supplementary table 1. Compared to patients with low eGFR, patients with high eGFR were younger (*p* = 0.029), had less frequently HT (*p* = 0.010) and had less frequently deceased donor (*p* = 0.024). Other characteristics were similar between groups.

### Relationship between dipping status and eGFR at T1: cross-sectional analysis

Proteinuria required log-transformation in regression analyses. In univariate analysis, dipping status was positively associated with eGFR at T1 (*p* = 0.013) and compared to non-dippers, dippers had a 9.4 mL/min/1.73m^2^ higher eGFR. In multivariate analysis, dipping status was positively associated with eGFR (*p* = 0.019) and compared to non-dippers, dippers had a 10.1 mL/min/1.73m^2^ higher eGFR (Table 3). Age and proteinuria were negatively associated with eGFR (*p* = 0.018 and 0.002 respectively). Other covariates were not associated with eGFR.

**Table 3:** Factors associated with eGFR at T1 in multivariable linear regression

**Independent variables**	**Final model**		
	**β**	**95% CI**	***p***
Systolic dipping status	10.13	1.70 to 18.55	**0.019**
Gender (woman)	6.82	-1.64 to 15.29	0.11
Age (years)	-0.33	-0.61 to -0.06	**0.018**
BMI (kg/m^2^)	0.68	-0.34 to 1.71	0.18
Smoking	4.89	-5.25 to 15.04	0.34
Hypertension^a^	-10.09	-21.87 to 1.68	0.09
Diabetes^a^	4.08	-4.97 to 13.14	0.37
Graft vintage^b^ (years)	-1.07	-2.48 to 0.33	0.13
Proteinuria (g/day)^c^	-7.52	-12.09 to -2.94	**0.002**
Deceased donor	-5.52	-13.64 to 2.59	0.18
Past rejection	-5.22	-16.04 to 5.60	0.34
CNI	-1.88	-18.19 to 14.42	0.81
Prednisone	-2.12	-10.80 to 6.55	0.62
RAA blocker	6.09	-2.79 to 14.97	0.17

*Abbreviations*: *eGFR* Estimated glomerular filtration rate, *BMI* Body mass index, *CNI* Calcineurin inhibitor, *RAA* Renin angiotensin aldosterone

^a^Defined based on related medication

^b^Corresponding to the timespan between T0 and T1

^c^Log transformed

### Relationship between dipping status and eGFR over time: longitudinal analysis

**Fig. 1:**
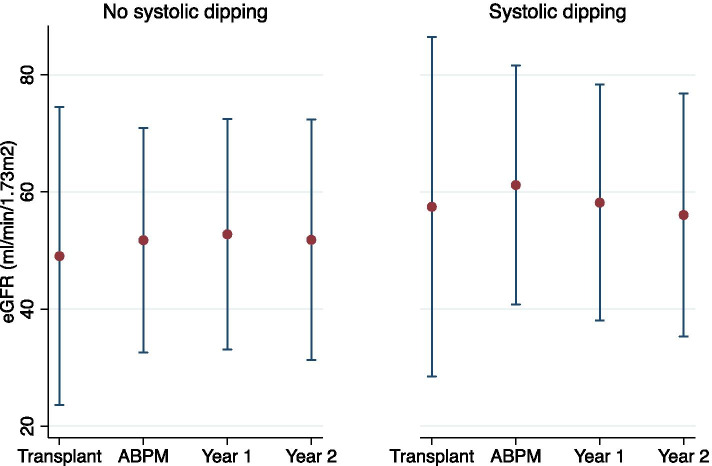
Evolution of eGFR over time according to systolic dipping status in univariate analysis. Dots and whiskers represent mean and standard deviation respectively. Abbreviations: eGFR, estimated glomerular filtration rate; SD, standard deviation

Mean follow-up after ABPM was 2.12 ± 0.45 years. In univariate analysis, dipping status was positively associated with eGFR (*p* = 0.012) and compared to non-dippers, dippers had a 9.1 mL/min/1.73^2^ higher eGFR. An interaction between time and dipping status was present (*p* = 0.085 for LRT): eGFR slope was -0.3 mL/min/1.73m^2^/year in non-dippers (*p* = 0.59) and -2.4 mL/min/1.73m^2^/year in dippers (*p* = 0.010). eGFR evolution over time between dippers and non-dippers is illustrated in Fig. 1. In multivariate analysis, dipping status was positively associated with eGFR (*p* = 0.009) and compared to non-dippers, dippers had a 10.48 mL/min/1.73m^2^ higher eGFR (Table 4). Interaction between time and dipping status was not significant (*p* = 0.17 for LRT) and eGFR slopes were thus not different between dippers and non-dippers. HT was negatively associated with eGFR (*p* = 0.003). Other covariates were not associated with eGFR.

**Table 4:** Factors associated with eGFR over time in multivariable mixed linear regression

**Independent variables**	**Final model**		
	**β**	**95% CI**	***p***
Systolic dipping status	10.48	2.63 to 18.32	**0.009**
Time (years)	0.43	-1.17 to 2.05	0.59
Dipping*time^a^	-1.86	-4.52 to 0.80	0.17
Gender (woman)	3.85	-3.85 to 11.56	0.32
Age (years)	-0.20	-0.46 to 0.05	0.11
BMI (kg/m^2^)	0.00	-0.76 to 0.78	0.98
Smoking	5.00	-4.97 to 14.99	0.32
Hypertension^b^	-12.59	-20.98 to -4.20	**0.003**
Diabetes^b^	0.72	-5.20 to 6.65	0.81
Graft vintage^c^ (years)	-0.72	-1.91 to 0.46	0.23
Proteinuria (g/day)^d^	0.02	-1.93 to 1.98	0.98
Deceased donor	-2.27	-9.54 to 5.00	0.54
Past rejection	-1.81	-9.05 to 5.42	0.62
CNI	0.91	-7.01 to 8.84	0.82
Prednisone	-3.53	-7.75 to 0.68	0.10
RAA blocker	-0.28	-3.77 to 3.21	0.87

*Abbreviations*: *eGFR* Estimated glomerular filtration rate, *BMI* Body mass index, *CNI* Calcineurin inhibitor, *RAA* Renin angiotensin aldosterone

^a^Interaction term between systolic dipping status and elapsed time

^b^Defined based on related medication

^c^Corresponding to the timespan between T0 and T1

^d^Log transformed

### Sensitivity analyses

HT defined by ABPM values instead of related medication was substituted in the final multivariate model. Dipping status remained positively associated with eGFR (*p* = 0.005). HT based on ABPM definition was negatively associated with eGFR (*p* = 0.019).

Diastolic and mean dipping status instead of systolic dipping status were substituted in the final multivariate model. Mean and diastolic dipping statuses were positively associated with eGFR (*p* = 0.029 and *p* = 0.010 respectively).

Systolic dip as continuous variable instead of systolic dipping as a binary variable was substituted in the final multivariate model. Systolic dip was not associated with eGFR (*p* = 0.06).

## Discussion

In this longitudinal study, we described circadian BP patterns in KTX recipients, compared OBPM and ABPM value in this setting and identified systolic dipping status as a major determinant of kidney function.

In our cohort, prevalence of HT when combining OBPM and ABPM measurement was 82.6%. Prevalence of HT based on ABPM only was 71.9%. Those numbers are in agreement with previous studies where HT has been reported to affect as many as 80% of KTX recipients depending on BP measurement modality [22]. Non-dipping status was present in 65.8% of our patients. Again, similar studies found comparable results [12, 14]. Systolic, diastolic and mean dipping statuses were well-correlated and systolic dippers tended to be mean and diastolic dippers as well. Finally, dipping status was not associated with the presence of HT on ABPM in our cohort and dippers were as likely as non-dippers to have HT. This absence of a direct relationship between HT and dipping status was also highlighted in CKD patients where non-dippers with controlled BP were almost as prevalent as non-dippers with HT [8].

Globally, systolic dipping status and the presence of HT were independently associated with eGFR in our study on KTX recipients, while other traditional determinants of kidney function were not as important.

### Relationship between dipping status and kidney function

Circadian BP patterns have been extensively studied in non-KTX patients. In 48 HT CKD patients followed by Timio et al., non-dippers had faster rates of renal function decline and higher proteinuria compared to dippers over a 3-year follow-up [23]. In 322 patients referred for ABPM, Davidson et al. found that eGFR remained stable among dippers over a 3.2 year follow-up but declined among non-dippers independently of SBP load [4]. The same year, Agarwal et al. reported on 217 CKD patients followed during 3.5 years where non-dipping status was an independent predictor of end-stage renal disease [24]. Finally, McMullan et al. found that nocturnal dipping was associated with a decreased risk of incident CKD over a 8.1 year follow-up in 603 Afro-American patients with normal renal function [25]. As these studies globally concluded that dipping status was an independent determinant of renal function over time, some evidences suggest that such a relationship does not exist. As such, Gabbai et al. noted that, although 24 h SBP was associated with subsequent renal and CV outcomes, dipping status did not per se predict progression of renal disease in 617 Afro-American with HT CKD [26]. Likewise, two similar successive studies were published by another group enrolling 676 and 1′107 CKD patients with two and 4.7-year follow-up periods respectively [6, 8]. Authors generally concluded that non-dipping pattern with normotension did not predict the future incidence of renal outcomes.

In KTX patients, description of BP patterns is much more sparse. Successful KTX is generally thought to improve circadian BP profile in the long term [27, 28]. The impact of these profiles and renal function is however less well defined. In an early study, Kooman et al. described a relationship between nightly decrease in MAP and kidney function in 36 renal recipients [15]. Cross-sectional design and absence of adjustment for potential confounders however hampered conclusions. Haydar et al. conducted a similar study on 177 patients where SBP circadian variation was associated with eGFR [16]. The same limitations were found although a limited set of covariates was considered. Later, Wadei et al. described an association between nocturnal fall in SBP and eGFR at 1 year after KTX in 119 patients [17]. This study was however cross-sectional in nature and very few potential confounders were considered. The same group focused on a sub-group of 36 of these patients who had a 3 to 4-year follow-up [29]. They found that the importance of nocturnal fall in SBP at 1 year was related to eGFR at last follow-up while adjusting for donor age and office SBP.

The main finding of our study is the strong and independent association between preserved systolic dipping status and improved renal function in KTX patients over a 2-year follow-up. Compared to previous publications on KTX patients, our study differs on several aspects. First, owing to the longitudinal design, we implemented multi-level mixed effect analysis in order to account for repeated collection of data. By adding subject-specific random intercept and slope effects to the population average, these models permit quantification of subject heterogeneity [30]. Moreover, this methodology allows comparison of eGFR slopes, recently recognized as a valid surrogate end point and likely more useful than time-to-event analysis for short follow-up with high baseline eGFR [31]. Second, an extensive set of potential confounders was a priori selected for multivariate analysis. This is of prime importance in this field as several factors, particularly BP control and proteinuria, were shown to confound the intricate relationship between dipping status and renal function. As an example, Agarwal et al. found that non-dipping status was predictive of CV events in a prospective cohort of 217 CKD patients [7]. However, this relationship disappeared when adjusted for proteinuria or clinic BP. In our study, a preserved dipping status was associated with a higher eGFR independently of potential confounders. The presence of HT was the only other significant determinant of renal function in longitudinal analysis. However, it is worth noting that not only did dipping status maintain its association with renal function beyond intensity of BP control but also its effect size on eGFR was comparable to that of HT itself. A recent study by Mallamaci et al. was designed similarly to ours as the authors reported on the impact of baseline ABPM on subsequent renal function over a 3.7-year follow-up in a cohort of 260 KTX recipients [18]. In multivariate analysis, 24 h, daytime and night-time absolute BP values were negatively associated with eGFR. However, this crude description of main ABPM components gave no specific information about a potential impact of circadian BP patterns or dipping status. Moreover, although presented analyses account for elapsed time, eGFR slopes are not depicted. The purpose of this study was thus rather different than ours and results are not directly comparable.

Finally, in previous studies, whether alteration in circadian BP profile was the cause or the consequence of kidney function decline was not entirely clear. In our study, although dippers had higher eGFR compared to non-dippers overall, differences seemed most striking at T1 when ABPM was recorded. eGFR slope analysis confirmed that dippers had a faster rate of function decline compared to non-dippers in univariate analysis. When considering potential confounders however, kidney function decline rates were similar between groups. This type of time trajectory is in agreement with the underlying physiological assumption that correlation between dipping status on a single ABPM assessment and induced TOD should be maximal at the time of initial measurement to then decrease during follow-up owing to the low reproducibility of circadian patterns over time [32]. As a matter of fact, any inter-group comparison in a follow-up study is based on dipping classification defined at baseline ABPM that does not necessarily represent actual dipping status at later time points. In a previous study on CKD patients with established diabetic nephropathy, time trajectories of renal function according to dipping status were similar to ours [33]. Globally, those findings are thus in favour of a causal effect of dipping status on renal function and not the other way around. It has to be noted that Wadei et al. described the opposite phenomenon in their longitudinal study where the association between dipping status and renal function was inexistent at initial evaluation but became significant during follow-up [29]. This apparent paradox is however not incompatible with our results. First, compared to our study, minimal adjustment only was considered and those results could represent residual confounding. Second, ABPM was conducted 1-year post transplant in their cohort while median graft vintage at ABPM was 2.5 years in our study. Thus, the longer time span between transplant and initial evaluation in our study could have allowed sufficient influence of established circadian BP patterns to impact renal function.

### Limitations

Our study has limitations that should be considered when interpreting the results. First, the observational nature of the design hampers definitive conclusions on causal relationship between considered variables. Namely, as the interplay between dipping status (and more generally BP control) and renal function is intricate, reverse causality should be considered. While the longitudinal design, statistical methodology and pathophysiologic considerations could indicate a causal effect of circadian BP patterns on renal function, a reverse impact of eGFR on dipping status is possible. Second, as in most similar studies, a single ABPM measurement was conducted. Longitudinal evaluation could thus be hampered by the intrinsic moderate reproducibility of dipping categorization. We believe however that our findings were discussed in the light of this phenomenon. Third, the rather limited sample size prevented us to further investigate specific sub-group of patients and to detect potential association with smaller effect size. However, achieved statistical power was by definition sufficient to investigate our primary objective as attested by the highly significant and robust associations presented. Fourth, as a fraction only of screened KTX patients had ABPM performed, selection bias cannot be excluded. Excluded patients were however similar to those included. Finally, the number of donor characteristics considered in our study was limited, as ethics regulation does not allow documentation of such data in medical records at our institution.

## Conclusion

In conclusion, we confirm that non-dipping status is highly prevalent in KTX patients while not necessarily associated with the presence of HT. Moreover, we suggest that systolic dipping status could be a major determinant of renal function in KTX patients. This relationship was independent of major potential confounders, including HT and proteinuria. The only other determinant of renal function was the presence of HT itself, in agreement with prior knowledge in non-KTX patients. Post-transplant ABPM should be offered to all KTX recipients as a screening tool, even in those with controlled OBPM. Whether modification of dipping status by chronotherapy would preserve renal function cannot be inferred from our results and remains to be tested in clinical trials.

## Supplementary Information

**Additional file 1: Supplementary table 1.** Patients characteristics according to median eGFR value at T1.

## Data Availability

The datasets used and/or analysed during the current study are available from the corresponding author on reasonable request.

## Abbreviations

ABPM: Ambulatory blood pressure monitoring
BMI: Body mass index
BP: Blood pressure
CI: Confidence interval
CKD: Chronic kidney disease
CV: Cardiovascular
DBP: Diastolic blood pressure
eGFR: Estimated glomerular filtration rate
HT: Hypertension
LRT: Likelihood ratio test
MAP: Mean arterial pressure
OBPM: Office blood pressure measurement
RRI: Renal resistive index
KTX: Kidney transplant
SBP: Systolic blood pressure
SD: Standard deviation
TOD: Target organ damage
US: Ultrasound

## References

[1] Banegas JR, Ruilope LM, de la Sierra A, Vinyoles E, Gorostidi M, de la Cruz JJ (2018). Relationship between clinic and ambulatory blood-pressure measurements and mortality. N Engl J Med.

[2] Gaborieau V, Delarche N, Gosse P (2008). Ambulatory blood pressure monitoring versus self-measurement of blood pressure at home: correlation with target organ damage. J Hypertens.

[3] Minutolo R, Agarwal R, Borrelli S, Chiodini P, Bellizzi V, Nappi F (2011). Prognostic role of ambulatory blood pressure measurement in patients with nondialysis chronic kidney disease. Arch Intern Med.

[4] Davidson MB, Hix JK, Vidt DG, Brotman DJ (2006). Association of impaired diurnal blood pressure variation with a subsequent decline in glomerular filtration rate. Arch Intern Med.

[5] Jaques DA, Müller H, Martinez C, De Seigneux S, Martin PY, Ponte B (2018). Nondipping pattern on 24-h ambulatory blood pressure monitoring is associated with left ventricular hypertrophy in chronic kidney disease. Blood Press Monit.

[6] Kado H, Kusaba T, Matoba S, Hatta T, Tamagaki K (2019). Normotensive non-dipping blood pressure profile does not predict the risk of chronic kidney disease progression. Hypertens Res.

[7] Agarwal R, Andersen MJ (2006). Blood pressure recordings within and outside the clinic and cardiovascular events in chronic kidney disease. Am J Nephrol.

[8] Ida T, Kusaba T, Kado H, Taniguchi T, Hatta T, Matoba S (2019). Ambulatory blood pressure monitoring-based analysis of long-term outcomes for kidney disease progression. Sci Rep.

[9] Opelz G, Wujciak T, Ritz E (1998). Association of chronic kidney graft failure with recipient blood pressure. Kidney Int.

[10] Weir MR, Burgess ED, Cooper JE, Fenves AZ, Goldsmith D, McKay D (2015). Assessment and management of hypertension in transplant patients. J Am Soc Nephrol.

[11] Mange KC, Cizman B, Joffe M, Feldman HI (2000). Arterial hypertension and renal allograft survival. J Am Med Assoc.

[12] Farmer CKT, Goldsmith DJA, Cox J, Dallyn P, Kingswood JC, Sharpstone P (1997). An investigation of the effect of advancing uraemia, renal replacement therapy and renal transplantation on blood pressure diurnal variability. Nephrol Dial Transplant.

[13] Kendirlinan Demirkol O, Oruc M, Ikitimur B, Ozcan S, Gulcicek S, Soylu H (2016). Ambulatory blood pressure monitoring and echocardiographic findings in renal transplant recipients. J Clin Hypertens.

[14] Covic A, Segall L, Goldsmith DJA (2003). Ambulatory blood pressure monitoring in renal transplantation: should ABPM be routinely performed in renal transplant patients?. Transplantation.

[15] Kooman JP, Christiaans MHC, Boots JMM, Van der Sande FM, Leunissen KML, Van Hooff JP (2001). A comparison between office and ambulatory blood pressure measurements in renal transplant patients with chronic transplant nephropathy. Am J Kidney Dis.

[16] Haydar AA, Covic A, Jayawardene S, Agharazii M, Smith E, Gordon I (2004). Insights from ambulatory blood pressure monitoring: diagnosis of hypertension and diurnal blood pressure in renal transplant recipients. Transplantation.

[17] Wadei HM, Amer H, Taler SJ, Cosio FG, Griffin MD, Grande JP (2007). Diurnal blood pressure changes one year after kidney transplantation: relationship to allograft function, histology, and resistive index. J Am Soc Nephrol.

[18] Mallamaci F, D’Arrigo G, Tripepi R, Leonardis D, Porto G, Testa A (2018). Office, standardized and 24-h ambulatory blood pressure and renal function loss in renal transplant patients. J Hypertens.

[19] Williams B, Mancia G, Spiering W, Rosei EA, Azizi M, Burnier M (2018). 2018 ESC/ESH Guidelines for themanagement of arterial hypertension. Eur Heart J.

[20] Levey AS, Stevens LA, Schmid CH, Zhang Y, Castro AF, Feldman HI (2009). A new equation to estimate glomerular filtration rate. Ann Intern Med.

[21] Jaques DA, Davenport A (2020). High-flow arteriovenous fistula is not associated with increased extracellular volume or right ventricular dysfunction in haemodialysis patients. Nephrol Dial Transplant.

[22] Mangray M, Vella JP (2011). Hypertension after kidney transplant. Am J Kidney Dis.

[23] Timio M, Venanzi S, Lolli S, Lippi G, Verdura C, Monarca C (1995). “Non-dipper” hypertensive patients and progressive renal insufficiency: a 3-year longitudinal study. Clin Nephrol.

[24] Agarwal R, Andersen MJ (2006). Prognostic importance of ambulatory blood pressure recordings in patients with chronic kidney disease. Kidney Int.

[25] McMullan CJ, Hickson DA, Taylor HA, Forman JP (2015). Prospective analysis of the association of ambulatory blood pressure characteristics with incident chronic kidney disease. J Hypertens.

[26] Gabbai FB, Rahman M, Hu B, Appel LJ, Charleston J, Contreras G (2012). Relationship between ambulatory BP and clinical outcomes in patients with hypertensive CKD. Clin J Am Soc Nephrol.

[27] Gatzka CD, Schobel HP, Klingbeil AU, Neumayer HH, Schmieder RE (1995). Normalization of circadian blood pressure profiles after renal transplantation. Transplantation.

[28] Covic A, Gusbeth-Tatomir P, Mardare N, Buhaescu I, Goldsmith DJA (2005). Dynamics of the circadian blood pressure profiles after renal transplantation. Transplantation.

[29] Wadei HM, Amer H, Griffin MD, Taler SJ, Stegall MD, Textor SC (2011). Abnormal circadian blood pressure pattern 1-year after kidney transplantation is associated with subsequent lower glomerular filtration rate in recipients without rejection. J Am Soc Hypertens.

[30] Shou H, Hsu JY, Xie D, Yang W, Roy J, Anderson AH (2017). Analytic considerations for repeated measures of eGFR in cohort studies of CKD. Clin J Am Soc Nephrol.

[31] Levey AS, Gansevoort RT, Coresh J, Inker LA, Heerspink HL, Grams ME (2020). Change in albuminuria and GFR as end points for clinical trials in early stages of CKD: a scientific workshop sponsored by the National Kidney Foundation in collaboration with the US Food and Drug Administration and European Medicines Agency. Am J Kidney Dis.

[32] James MA, Fotherby MD, Potter JF (1995). Reproducibility of the circadian systolic blood pressure variation in the elderly. J Hypertens.

[33] Farmer CKT, Goldsmith DJA, Quin JD, Dallyn P, Cox J, Kingswood JC (1998). Progression of diabetic nephropathy - Is diurnal blood pressure rhythm as important as absolute blood pressure level?. Nephrol Dial Transplant.

