# Supplementary material for: Relationship between renal function and blood pressure dipping status in renal transplant recipients: a longitudinal study
Source: BMC Nephrol. 2021 Sep 30;22:325. doi: 10.1186/s12882-021-02523-7 (PMC8485467; doi:10.1186/s12882-021-02523-7)
Supplement: Supplementary file 1 — Additional file 1: Supplementary table 1. Patients characteristics according to median eGFR value at T1. [file 12882_2021_2523_MOESM1_ESM.docx]

**RELATIONSHIP BETWEEN RENAL FUNCTION AND BLOOD PRESSURE DIPPING STATUS IN RENAL TRANSPLANT RECIPIENTS: A LONGITUDINAL STUDY.**

David A. JAQUES, MD; Patrick SAUDAN, MD; Chantal MARTINEZ, Axel ANDRES, MD; Pierre-Yves MARTIN, MD; Antoinette PECHERE-BERTSCHI, MD; Belen PONTE, MD.

**SUPPLEMENTARY MATERIAL**

**Supplementary table 1:** Patients characteristics according to median eGFR value at T1.

| **Characteristics** | **Overall (N=126)** | **Low eGFR (N=63)** | **High eGFR (N=63)** | **P value** |
| --- | --- | --- | --- | --- |
| **Clinical characteristics** | | | | |
| **Age (years)** | 56.4 +/- 15.1 | 59.3 +/- 15.0 | 53.4 +/- 14.7 | **0.029** |
| **BMI (kg/m^2^)** | 25.8 +/- 4.0 | 26.0 +/- 4.2 | 25.5 +/- 3.9 | 0.47 |
| **Gender (female)** | 46 (36.5%) | 22 (34.9%) | 24 (38.1%) | 0.71 |
| **Smoking** | 21 (16.6%) | 10 (15.8%) | 11 (17.4%) | 0.81 |
| **Diabetes^a^** | 30 (24.0%) | 13 (20.6%) | 17 (27.4%) | 0.37 |
| **Hypertension^a^** | 107 (85.6%) | 59 (93.6%) | 48 (77.4%) | **0.010** |
| **Dyslipidemia^a^** | 71 (56.8%) | 40 (63.4%) | 31 (50.0%) | 0.12 |
| **IHD** | 18 (14.4%) | 12 (19.3%) | 6 (9.5%) | 0.11 |
| **PVD** | 13 (10.4%) | 9 (14.5%) | 4 (6.3%) | 0.13 |
| **OSA** | 18 (14.2%) | 9 (14.2%) | 9 (14.2%) | 1 |
| **Paraclinical characteristics** | | | | |
| **eGFR (mL/min/1.73m^2^)** | 54.7 +/- 19.9 | 39.1 +/- 10.9 | 70.3 +/- 13.5 | **<0.001** |
| **Proteinuria (g/day)** | 0.1 (0.1 – 0.3) | 0.2 (0.1 – 0.4) | 0.1 (0.1 – 0.2) | 0.06 |
| **RRI (%)** | 67.7 +/- 7.9 | 68.9 +/- 7.4 | 66.4 +/- 8.2 | 0.12 |
| **Transplant characteristics** | | | | |
| **Graft vintage (years)** | 2.4 (0.6 – 5.9) | 3.0 (0.5 – 7.5) | 1.9 (0.7 – 4.9) | 0.12 |
| **Deceased donor** | 68 (54.4%) | 40 (64.5%) | 28 (44.4%) | **0.024** |
| **Past rejection** | 23 (18.2%) | 14 (22.2%) | 9 (14.2%) | 0.24 |
| **Pre-transplant status**   - **Pre-emptive** - **PD** - **HD** | 28 (22.2%)  14 (11.1%)  84 (66.6%) | 9 (14.2%)  6 (9.5%)  48 (76.1%) | 19 (30.1%)  8 (12.7%)  36 (57.1%) | 0.06 |
| **Medication** | | | | |
| **CNI** | 120 (95.2%) | 61 (96.8%) | 59 (93.6%) | 0.40 |
| **Prednisone** | 78 (66.6%) | 43 (74.1%) | 35 (59.3%) | 0.08 |
| **RAA blocker^b^** | 44 (35.2%) | 26 (41.2%) | 18 (29.0%) | 0.15 |
| **FK level (ng/mL)^c^** | 8.0 +/- 3.0 | 8.2 +/- 3.3 | 7.8 +/- 2.7 | 0.42 |
| **CsA level (ng/mL)^d^** | 136.8 +/- 38.0 | 129.6 +/- 40.8 | 151.3 +/- 29.7 | 0.26 |

a: Defined based on related medication.

b: Angiotensin converting enzyme inhibitor or angiotensin II receptor blocker*.*

c: Available in a subgroup of 98 patients.

d: Available in a subgroup of 18 patients.

*Abbreviations: eGFR, estimated glomerular filtration rate; BMI, body mass index; IHD, ischemic heart disease; PVD, peripheral vascular disease; OSA, obstructive sleep apnoea; eGFR, estimated glomerular filtration rate; RRI, renal resistive index; PD, peritoneal dialysis; HD, haemodialysis; CNI, calcineurin inhibitor; RAA, renin angiotensin aldosterone; FK, tacrolimus, CsA, cyclosporine.*
